# Associations between digital financial services and health campaign workers’ perceived satisfaction, motivation and performance in Nigeria

**DOI:** 10.1136/bmjgh-2024-018384

**Published:** 2025-09-22

**Authors:** Folashayo Peter Adeniji, David Adewole, Segun Bello, Juliet Aweko, Elizabeth Ekirapa Kiracho, Peter Waiswa, Fredrick Makumbi, Olufunmilayo Fawole

**Affiliations:** 1 Department of Health Policy and Behavioral Sciences, Georgia State University—Atlanta Campus, Atlanta, Georgia, USA; 2 Health Policy and Management, University of Ibadan College of Medicine, Ibadan, Nigeria; 3 Department of Epidemiology and Medical Statistics, University of Ibadan College of Medicine, Ibadan, Oyo, Nigeria; 4 Department of Health Policy, Planning and Management, Makerere University School of Public Health, Kampala, Kampala, Uganda; 5 School of Public Health, Department of Health Policy, Planning and Management, Makerere University, Kampala, Uganda; 6 Epidemiology and Biostatistics, Makerere University, Kampala, Central Region, Uganda

**Keywords:** Health Personnel, Global Health, Health policies and all other topics, Health policy

## Abstract

**Background:**

Understanding the association between digital payment services and health worker outcomes is critical to improving public health outcomes and optimising health workforce efficiency. This study examined the link between digital payment systems and health campaign workers’ perceived satisfaction, motivation and performance in Nigeria.

**Methods:**

A national survey was conducted among 821 polio immunisation workers who participated in campaigns. A structured electronic questionnaire which had sections on sociodemographic characteristics and digital payments was used to elicit information from participants. Campaign workers’ self-reported satisfaction, motivation and performance were assessed using standardised tools for examining these outcomes. Linear mixed model regression analysis was used to examine the factors associated with health worker’s motivation, satisfaction and performance. Predictors of these outcomes during periods of cash and digital payment methods were assessed, with statistical significance set at the 5% level.

**Results:**

This cross-sectional study included 821 health campaign workers in Nigeria, with a mean age of 37.13 years (SD=11.06). Regarding digitised payments, mean scores across all constructs were generally high: general motivation (16.34±2.58; maximum=20), job satisfaction (20.33±3.16; max=25), intrinsic job satisfaction (12.87±1.62; max=15), organisational commitment (16.99±1.96; max=20), conscientiousness (15.68±1.71; max=20), timeliness and attendance (13.35±1.55; max=15) and perceived performance (20.54±2.97; max=25). Overall, the combined motivation, satisfaction and performance score was higher among workers paid via digital methods compared with those paid in cash (104.14±9.34 vs 68.43±50.75). In multivariable linear regression, being female (β=1.83; 95% CI=0.28 to 3.38) and the ability to use the internet (β=1.87; 95% CI=0.02 to 3.72) were significant independent predictors of higher overall scores.

**Conclusion:**

Digital payment methods were associated with higher health workers’ perceived job satisfaction, motivation and performance. Public health stakeholders need to promote the use of digital payments to maximise their positive influence on health workers.

**WHAT IS ALREADY KNOWN ON THIS TOPIC**

- Digital payment systems provide greater timeliness, transparency and efficiency in remunerating health campaign workers in low-income countries compared with cash payments.

**WHAT THIS STUDY ADDS**

- Digital payment of health campaign workers was associated with higher self-reported motivation, job satisfaction and performance.
- Use of digital financial services may contribute to improved workforce-related outcomes in health campaigns.
- Findings highlight the potential value of integrating digital payment systems to strengthen health campaign delivery in Nigeria

**HOW THIS STUDY MIGHT AFFECT RESEARCH, PRACTICE OR POLICY**

- Highlights the potential of electronic payment systems to strengthen the implementation of health programmes.
- Findings inform policy decisions, improve healthcare management strategies and provide useful information on how the integration of digital payment innovations can improve health campaign outcomes in low-income countries.

- Highlights the need for public health stakeholders to invest in digital payment systems and provide education for health campaign workers on their effective use

## Introduction

 The integration of digital technologies into various aspects of healthcare delivery has improved the payment process of health workers, especially in low-income countries.^1^,^4^ In many African countries, digital financial services (DFS) have been increasingly adopted to address the challenges associated with the use of physical cash to pay healthcare workers. Consequently, DFS is having a significant impact on the payment of health campaign workers, who play a critical role in promoting public health initiatives.^5^,^7^ Globally, health promotion programmes and campaigns are important strategies being used by governments to control diseases, promote immunisation and raise public awareness about health problems.^8^,^11^ A vital component of these efforts is the health workers who actively participate in programme delivery, which is critical to the success of these campaigns.^12^,^14^ Traditionally in many African countries, health workers’ payment systems were manual, prone to delay and lacked transparency. Unfortunately, this resulted in dissatisfaction, demotivation and a decline in work performance.^15 16^

The Nigerian healthcare system has increasingly embraced the use of digital payment services, recognising the need for a more efficient and transparent system.^17 18^ However, current mechanisms for payment of health workers often combine digital and cash payment methods, resulting in inefficiency with regards to the timeliness and completeness of their pay, including embarking on industrial action among health campaign workers.^7^ Therefore, transitioning to the exclusive use of digital payment methods can promote work efficiency and improve health outcomes.^19^ For instance, workers participating in health campaigns can be paid directly into their bank accounts or mobile wallets, thereby avoiding the delays and uncertainties often associated with traditional payment methods.^5 15 20^ Thus, the shift to digitised compensation has the potential to change the dynamics of health campaigns, ultimately leading to better public health outcomes.^5 8 16 19^

The satisfaction, motivation and performance of health campaign workers are critical factors influencing their commitment and dedication to their work.^14 21^ On-time payments enabled by digital payment services are likely to improve job satisfaction by providing financial stability and security.^21^ Additionally, the transparency and accountability built into digital payment systems can build trust between the healthcare authorities and campaign workers, thereby increasing the health workers’ job satisfaction.^22^ In the workplace, including during campaigns, motivation is also a key factor influencing performance. Thus, the adoption of digital payment services provides an opportunity to address the motivational challenges that Nigerian health campaign workers experience.^14 21 23 24^ By ensuring prompt and secure payments, digital payment systems have the potential to boost health workers’ motivation and morale, encouraging a more dedicated and enthusiastic approach to their tasks.^21 25^

With many health campaigns being implemented across Nigeria, understanding the impact of digital health payment services on health workers’ satisfaction, motivation and performance is critical as the Nigerian healthcare system strives to improve public health outcomes, while optimising workforce efficiency. This study examined the influence of digital payment systems on health campaign workers’ self-perceived satisfaction, motivation and performance compared with traditional cash payment methods. To the best of our knowledge, research addressing this gap in many low- and middle-income countries is limited.

## Methods

### Study setting

Nigeria is divided into six geopolitical zones and 36 states, with a total population of approximately 217 million people as at 2023.^26^ More than 60 million adults live in the rural areas, while about 35 million reside in the cities.^26^ Approximately half of the rural population lacks access to adequate digital payment infrastructure, such as reliable banking networks and internet connectivity, leaving them financially excluded, that is, unable to access or use basic financial services like bank accounts, digital payments or credit. In urban areas, about one-quarter of the adult population also remains financially excluded.^27^

In Nigeria, immunisation campaigns are managed by the National Primary Healthcare Development Agency in collaboration with the Federal and State Ministries of Health (SMoH). Implementing partners, such as the WHO, Global Alliance for Vaccine and Immunisation, UNICEF, Save the Children, the Clinton Health Access Initiative, the World Bank and the Bill & Melinda Gates Foundation and Aliko Dangote Foundation, provide financial and technical support for these campaigns. Typically, these partners work through the government health structures to plan, implement and monitor campaigns. For example, in polio eradication programmes, the WHO collaborates with SMoH to conduct environmental surveillance (eg, testing sewage samples for poliovirus) and organise targeted vaccination campaigns (‘mop-ups’). Additionally, SMoH are mandated to conduct supplemental immunisation activities periodically.

### Study design

This study adopted a cross-sectional survey design to examine the influence of digital and cash payment systems on campaign workers’ perceived satisfaction, motivation and performance.

### Study population

The study population consisted of health campaign workers engaged in immunisation campaigns. These workers included: vaccinators, team supervisors, recorders and social mobilisers. Health workers were recruited through the SMoH based on the following eligibility criteria: (1) they should have participated in at least a previous immunisation campaign and (2) should have received payment for work done either through digital means, cash or a combination of both. Workers who were not paid after participating in a campaign were excluded from the study, as the focus of the study was to compare experiences based on payment modalities.

### Sample size determination

The minimum sample size for the survey was estimated, using the formula for single proportion (Z^2^pq/d^2^), Z was the standard normal deviate corresponding to a 95% confidence level (1.96), p was the assumed proportion of the population with the outcome (ie, health workers’ perceived motivation, satisfaction and performance), q=1 p, and d was the acceptable margin of error (0.05). In the absence of prior studies assessing the composite outcome of perceived satisfaction, motivation and performance among health campaign workers, a conservative estimate of p=0.5 was used. This proportion is commonly adopted when prior data is unavailable, as it yields the maximum required sample size, thereby ensuring sufficient statistical power to detect meaningful associations in multivariable regression analysis. To adjust for the survey design and potential clustering effects, a design effect of 2 was applied. A minimum required sample size of 768 participants was obtained. After a 5% adjustment for non-response, the estimated sample size was 806 health campaign workers.

### Sample selection

Nigeria has six geopolitical zones; three in the North (North East, North Central and North West) and three in the South (South West, South South and South East). After stratifying into two based on north–south divide, four geopolitical zones were randomly selected: two from the north and two from the southern zones. In each of the selected zones (4), security and feasibility of safe data collection was taken into consideration, after which one (1) state was randomly selected. The selected states (4) included: the Federal Capital Territory, Akwa Ibom, Sokoto and Oyo. The sample size was proportionately distributed across the four selected states. Within each state, one urban and one rural local government area (LGA) was selected using a simple random sampling technique. In the absence of a sampling frame, local immunisation officers assisted in contacting all immunisation campaign workers in the LGA. These health workers were enrolled in the study consecutively until the required sample size was reached. Interviews were conducted at the local government designated immunisation centres. To address potential sources of bias, standardised questionnaires and interviewer training were employed to reduce interviewer and measurement bias. To minimise social desirability bias, participants were assured of confidentiality and informed that their responses would not affect their employment or future campaign participation.

### Data collection

The survey instrument was a structured, interviewer-administered electronic questionnaire designed using the REDCap survey platform. The questionnaire had sections on respondents’ sociodemographic characteristics, work history and on the health workers’ perceived satisfaction, motivation and performance. These outcomes were assessed by adapting a multidimensional tool for measuring health workers’ motivation, which was developed in a previous study.^28^ This tool assessed overall perceived motivation, satisfaction as a composite of the following constructs: (1) general motivation; for example, I feel motivated to work as hard as I can; (2) job satisfaction; for example, overall, I am very satisfied with the digital means used in paying my salary; (3) intrinsic job satisfaction; for example, I am satisfied with the opportunity to use my abilities in my job; (4) organisational commitment; for example, I find that my values and the objectives of the health programme are very similar; (5) conscientiousness; for example, I cannot be relied on by my colleagues at work; (6) timeliness and attendance; for example, I am punctual about coming to work; (7) performance; for example, I am able to meet my performance target every day at work.

16 research assistants (4 per state) were trained on quality data collection and research ethics. To ensure the validity of the data collection tool, a pretest was conducted on a different population of health campaign workers (n=30) from those used in the study. Subsequently, the tool was revised based on feedback received during the pretest. Research assistants conducted face-to-face interviews and entered participants’ responses directly into the structured electronic questionnaire. This helped to avoid missing data, as all the required fields (questions) and questionnaires were completely filled. The coauthors FPA, DA, SB and OF served as supervisors in each of the selected states. As English is the official language used in Nigeria, the data collection tool was developed in English, and therefore no linguistic validation was required. English is also the primary language of communication in workplaces and educational settings in the country, and healthcare workers selected for the study were able to understand and communicate effectively in English. Data collection was conducted between 5 June 2023 and 16 June 2023, and each interview lasted approximately 40 min.

### Primary outcome variables

1. *Health workers’ perceived motivation*

Health workers’ perceived motivation was defined as the enthusiasm, personal drive, energy level, tenacity, commitment and willingness to contribute to the realisation of the organisation goals.

1. *Health workers’ perceived satisfaction*

Health workers’ perceived satisfaction was defined as the feeling of happiness and fulfilment they expressed in relation to their job contributions to the goals of healthcare delivery and to organisational goals in general. It was also a measure of the behavioural and cognitive aspects of health workers to their jobs. Health workers’ motivation and satisfaction were measured using broad constructs, namely: general motivation, job satisfaction, organisational commitment, conscientiousness, timeliness and attendance. These were scored and dichotomised.

1. *Health worker perceived performance*

Performance was the achievement of clear, measurable and quantified objectives of assigned work/job. It was measured from estimating health workers’ self-reported ability to meet the daily target of eligible children to be vaccinated.

### Data management and analysis

Data was cleaned and composite variables were derived. In general, the tool had 28 items/questions which captured different constructs of health worker perceived motivation and satisfaction (online supplemental Material). Each item was expressed on a 5-point Likert scale, namely: strongly disagree, disagree, undecided, agree and strongly agree. Scores from 1 to 5 were applied (1—strongly disagree and 5—for strongly agree for positively worded questions and the reverse for negatively worded items). A higher score indicated a higher level of motivation.^28^ The internal reliability of the measures of perceived motivation, satisfaction and performance was assessed using Cronbach’s alpha (α=0.93). Following this, composite scores were computed for (1) responses for the period of digital payment and (2) responses for the period of cash payment. Tests of normality and homogeneity of variance were conducted to assess the distribution of outcome scores, including individual constructs, as well as the composite score. All scores were found to be normally distributed. Therefore, appropriate summary measures, such as frequency distributions and means, were used to report health workers’ motivation, job satisfaction and performance.

The student t-test was used to compare means of scores for health workers’ perceived motivation, satisfaction and performance between digital payment and cash payment periods. A linear mixed model (LMM) regression analysis was used to explore the determinants of health worker motivation, satisfaction and performance. This approach is robust for handling the correlation between repeated measures on the same individual, as participants’ responses regarding their experiences with cash payment may have influenced their responses to questions about digital payment.^29^ In the LMM, LGA was specified as a random intercept. This was done to account for the hierarchical structure of the data, where health workers were nested within LGAs. The inclusion of LGA as a random effect helps to capture unobserved heterogeneity and potential clustering at the LGA level, which could bias standard errors if ignored. Data were analysed using Stata V.18.

### Patient and public involvement

No patient or public involvement in the design and implementation of this study. This was not needed for the achievement of study objectives.

## Results

### Sociodemographic characteristics

A total of 821 immunisation campaign workers participated in the national survey, the mean age was 37.13±11.06 years (table 1). The number of health workers included in the survey was 169 (20.6%) in Abuja, 188 (22.9%) in Cross River, 212 (25.8) in Oyo and 252 (30.7%) in Sokoto states. The majority were females (599, 73.3%). More than half (447, 59.6%) resided in urban locations. Also, more than half (61.5%) were currently married and 552 (67.9%) had tertiary education. Close to half of the participants (351, 43.7%) were not regular health workers. The cadre of health workers surveyed included the community health extension workers (CHEW) (196, 24.4%), community health officers (149, 18.6%) and junior CHEW (76, 9.5%) (table 1). Of the study participants, 535 (65.5%) had received both digital and cash payments, while 282 (34.5%) had received digital payment only.

**Table 1:** Sociodemographic characteristics of immunisation campaign workers who participated in the survey (N=821)

Sociodemographic variables	Frequency	Percentage
Mean age±SD	37.13 ± 11.06	
Age group (years)		
≤20	29	3.5
21–30	236	28.8
31–40	267	32.6
41–50	180	22.0
51 and above	107	13.1
State		
Abuja	169	20.6
Cross River	188	22.9
Oyo	212	25.8
Sokoto	252	30.7
Location		
Rural	303	40.4
Urban	447	59.6
Sex		
Male	218	26.7
Female	599	73.3
Marital status		
Currently married	500	61.5
Currently not married	313	38.5
Level of education		
No formal	4	0.5
Primary	29	3.6
Secondary	228	28.0
Tertiary	552	67.9
Cadre		
Nurse	32	4.0
Community health officer	272	33.5
Community health extension worker	233	28.7
Junior community health extension worker	113	13.9
*Others	161	19.9
Ever participated in vaccine campaign		
Yes	819	100.0
Mode of payment for work done on vaccination		
Both cash and digital	535	65.5
Digital only	282	34.5
Mode of notification of payment for work done		
SMS (yes)	787	95.9
Mobile wallet alert (yes)	134	16.3
Check bank balance (yes)	80	9.7
Email (yes)	38	4.6
Visit bank (yes)	38	4.6
Years of work experience		
<2	48	5.9
2–5	253	31.1
6–10	261	32.1
More than 10	252	31.0
Region of residence		
North Central	148	18.2
North West	253	31.1
South East	21	2.6
South-South	183	22.5
South West	209	25.7
Own a mobile		
No	12	1.5
Yes	807	98.5
Can access internet through personal mobile phone		
No	131	16.4
Yes	670	83.6
Able to use the internet		
No	137	16.8
Yes	679	83.2

* Others were social workers, health assistant/technician, community mobilisers, public health personnel/health educator, volunteer workers, environmental health officer/attendant.

The notification of payments was mainly through text messages (787, 95.9%), mobile wallet alert (134, 16.3%) and physical visit to the bank to check the account balance (80, 9.7%) (table 1). Other methods of notification were: email (38, 4.6%) and physical visit to the bank (38, 4.6%). About a third of participants had 2–5 years of work experience (253, 31.1%), 6–10 years of work experience was by (261) 32.1%, while more than 10 years of work experience was by (253) 31.1%. Almost all respondents (807, 98.5%) owned a mobile phone, 670 (83.6%) had access to mobile internet and 679 (83.2%) were able to use the internet (β=1.87; 95% CI=0.02 to 3.72).

### Health workers’ perceived motivation, satisfaction and performance while being paid with digital methods

Health workers’ perceived motivation, satisfaction and performance with digital payment during campaigns is shown in table 2. For the general motivation construct, the majority of the health workers either ‘strongly agreed’ or ‘agreed’ on their level of motivation with the digital method of payment. About 346 (42.1%) ‘strongly agreed’ and 449 (54.7%) ‘agreed’, on their ability to work very hard during digital mode of payment. A similar positive response was obtained under the ‘general motivation’ heading such as, ‘motivation to work due to the receipt of full and timely payment. However, 104 (12.7%) health workers ‘disagreed’ with the statement that they were worried when payment was made through bank transfers. Also, 109 (13.3%) ‘disagreed’ that payments were timely when digital platforms were used. The respondents’ responses were similar when observed for other constructs under health worker motivation, satisfaction and performance. The majority of the health workers either ‘strongly agreed’ or ‘agreed’ that they had job satisfaction (table 2). The mean scores across all constructs were positive, high and as follows: general motivation (16.34±2.58; maximum 20), job satisfaction (20.33±3.16; maximum 25), intrinsic job satisfaction (12.87±1.62; maximum 15), organisational commitment (16.99±1.96; maximum 20), conscientiousness (15.68±1.71; maximum 20), timeliness and attendance (13.35±1.55; maximum 15) and performance (20.54±2.97; maximum 25).

**Table 2:** Health workers’ perceived motivation, satisfaction and performance during digital payment

Domain	Strongly disagree	Disagree	Undecided	Agree	Strongly agree
General perceived motivation					
I feel motivated to work as hard as I can	4 (0.5)	21 (2.6)	1 (0.1)	449 (54.7)	346 (42.1)
I do not have to worry about the payment for vaccination campaign because it is paid as and when due using bank transfer	31 (3.8)	104 (12.7)	10 (1.2)	476 (58.1)	199 (24.3)
I am motivated to work because with the mode of payment, I am paid in full	8 (1.0)	44 (5.4)	14 (1.7)	475 (57.9)	280 (34.1)
Due to timeliness of receiving payment for vaccination campaign, I am always motivated to do my best	21 (2.6)	109 (13.3)	13 (1.6)	437 (53.2)	241 (29.4)
*Mean general motivation*	Mean (SD)=16.34 (2.58); Min=4; Max=20				
Perceived Job satisfaction					
Overall, I am very satisfied with the digital means used in paying me for vaccination campaign	7 (0.9)	42 (5.1)	9 (1.1)	392 (47.8)	370 (45.1)
I am satisfied with the time I get paid for vaccination campaign	41 (5.0)	195 (23.8)	18 (2.2)	375 (45.7)	192 (23.4)
I am not satisfied with means of paying me for vaccination campaign	236 (28.8)	378 (46.04)	28 (3.4)	143 (17.4)	36 (4.4)
I am satisfied with my work during vaccination campaign	3. (0.4)	6 (0.7)	2 (0.2)	459 (55.9)	351 (42.8)
The fact that I am paid through digital means has made me more satisfied with job	7 (0.9)	40 (4.9)	23 (2.8)	399 (48.7)	350 (42.7)
*Mean job satisfaction*	Mean (SD)=20.33 (3.16); Min=9; Max=25				
Perceived intrinsic job satisfaction					
I am satisfied with the opportunity to use my abilities during vaccination campaigns	1 (0.1)	1 (0.1)	3 (0.4)	448 (54.7)	366 (44.7)
I am satisfied that I accomplish something worthwhile during vaccination campaigns	4 (0.5)	7 (0.9)	6 (0.7)	478 (58.2)	326 (39.7)
I do not think that my work during vaccination campaigns is valuable in recent times	338 (41.2)	353 (43.0)	27 (3.3)	75 (9.1)	28 (3.4)
*Mean intrinsic job satisfaction*	Mean (SD)=12.87 (1.62); Min=7; Max=25				
Perceived organisational commitment					
I am proud to have worked on the vaccination campaign	0	3 (0.4)	1 (0.1)	382 (46.5)	435 (53.0)
I find that my values and the objectives of the vaccination campaign are similar	0	14 (1.7)	10 (1.2)	486 (59.2)	311 (37.9)
I am glad that I work on the vaccination campaign rather than other health programmes	10 (1.2)	50 (6.1)	34 (4.1)	488 (59.4)	239 (29.11)
I feel very little commitment to achieving the objectives of the vaccination campaign	306 (37.4)	386 (47.2)	37 (4.5)	72 (8.8)	17 (2.2)
*Mean organisational commitment*	Mean (SD)=16.99 (1.96); Min=11; Max=20				
Perceived conscientiousness					
I cannot be relied on by my colleagues during the vaccination campaign	338 (41.2)	353 (43.1)	17 (2.1)	83 (10.1)	29 (3.5)
I always complete my tasks efficiently and correctly during vaccination campaign	2 (0.2)	2 (0.2)	2 (0.2)	413 (50.4)	400 (48.8)
I am a hard worker during vaccination campaign	2 (0.2)		3 (0.4)	330 (40.2)	485 (59.2)
I do things that need doing without being asked or told during vaccination campaigns	3 (0.4)	39 (4.8)	5 (0.6)	396 (48.3)	377 (46.0)
*Mean conscientiousness*	Mean (SD)=15.68 (1.71); Min=4; Max=19				
Perceived timeliness and attendance					
I am punctual about coming to work during vaccination campaigns	0	2 (0.2)	2 (0.2)	332 (40.5)	484 (59.0)
I am often absent from work during vaccination campaigns	418 (51.0)	366 (44.6)	5 (0.6)	29 (3.5)	2 (0.2)
It is not a problem if I sometimes come late to work during vaccination campaigns	413 (50.5)	354 (43.3)	8 (1.0)	33 (4.0)	10 (1.2)
*Mean timeliness and attendance*	Mean (SD)=13.35 (1.55); Min=0; Max=15				
Perceived performance					
I am paid in full and in a timely manner during vaccination campaigns and this has improved my performance	14 (1.7)	78 (9.6)	24 (2.9)	445 (54.6)	254 (31.2)
Paying through digital means has not improved my job performance during vaccination campaigns	184 (22.6)	419 (51.4)	39 (4.8)	138 (16.9)	36 (4.4)
I am able to meet my performance target every day at work during vaccination campaigns	3 (0.4)	12 (1.5)	4 (0.5)	450 (55.1)	348 (42.6)
My job during vaccination campaigns has improved in recent times	1 (0.1)	8 (1.0)	5 (0.6)	500 (61.4)	301 (36.9)
My job during vaccination campaigns has not improved	309 (37.9)	438 (53.7)	9 (1.1)	50 (6.1)	9 (1.1)
*Mean performance*	Mean (SD)=20.54 (2.97); Min=0; Max=25				
Overall perceived motivation, satisfaction and performance	Mean (SD)=104.14 (9.34); Min=58, Max=122; Median (Q1, Q3)=104 (97, 111)				

### Health workers’ perceived satisfaction, motivation and performance (digital vs cash)

The test of difference in mean scores of different constructs of perceived motivation, satisfaction and performance for digital and cash payment systems is shown in table 3. Across all constructs, the mean scores were higher for digital payment compared with cash payment. The differences in means were statistically significant for all constructs. However, the differences were lowest for timeliness and attendance (15.68±1.71 vs 13.18±1.68) and organisational commitment (16.99±1.96 vs 16.01±3.19).

**Table 3:** Test of difference in perceived motivation, satisfaction and performance of health workers during vaccination campaigns (digital vs cash payments)

Constructs	Mean±SD (digital)	Mean/SD (cash)	t-statistic; p value
General motivation	16.34±2.58	12.71±4.29	16.32; <0.01
Job satisfaction	20.33±3.16	15.01±4.73	20.47; <0.01
Intrinsic job satisfaction	12.96±1.62	11.92±2.51	10.51; <0.01
Organisational commitment	16.99±1.96	16.01±3.19	8.41; <0.01
Conscientiousness	15.68±1.71	16.65±2.92	7.36; <0.01
Timeliness and attendance	13.35±1.55	13.18±1.68	4.12; <0.01
Performance	20.54±2.97	17.76±3.64	18.32; <0.01
Overall motivation	104.14±9.34	68.43±50.75	20.46; <0.01
Self-rated satisfaction	8.49 ± 1.71	5.96 ± 2.71	18.47; <0.01
Self-rated motivation	8.64 ± 1.63	6.12 ± 2.58	19.22; <0.01
Self-rated ability to meet daily target	8.73 ± 1.34	6.73 ± 2.88	15.45; <0.01
Self-rated overall job performance	8.82 ± 1.35	6.83 ± 2.71	15.91; <0.01

### Factors associated with overall health workers’ perceived motivation, satisfaction and performance at univariate level

Table 4 shows the univariate LMM of the overall perceived motivation, satisfaction and performance during the digital payment period. For workers who were paid through digital means, residing in urban locations (β=1.89, 95% CI=0.36 to 3.43) was associated with overall health workers’ motivation, satisfaction and performance, indicating that perceived motivation, satisfaction and performance of health workers residing in urban settings was higher than those living in rural locations by almost two points on the scale. Also, being female (β=3.98, 95% CI=2.29 to 5.66), being not married (β=1.64; 95 CI=0.11 to 3.17) and being paid by only digital means (β=−2.26, 95 CI=−3.86 to −0.67), were associated with (overall) health workers’ perceived motivation, satisfaction and performance among participants who were paid digitally. Also, health workers living in the North-West zone (β=10.67; 95% CI=8.80 to 12.54), South-East zone (β=19.81, 95% CI=15.53 to 24.09), South-South zone (β=14.26, 95% CI=12.26 to 16.25), South-West zone (β=11.76, 95% CI=9.65 to 13.86), had higher health workers’ perceived motivation, satisfaction and performance compared with those living in the North Central zone. Health workers who were able to use the internet had higher perceived motivation, satisfaction and performance compared with those who could not (β=4.10, 95% CI=2.06 to 6.15).

**Table 4:** Univariate linear mixed model of overall health workers’ perceived motivation, satisfaction and performance during digital payment

			95% CI		
	**β**	SE	**Lower**	**Upper**	**P value**
Age group (years)					
≤20	ref				
21–30	−0.44	2.11	−4.58	3.70	0.835
31–40	−0.41	2.11	−4.55	3.72	0.843
41–50	0.11	2.17	−4.14	4.38	0.956
51 and above	1.09	2.28	−3.39	5.58	0.632
Location					
Rural	ref				
Urban	1.89	0.78	0.36	3.43	**0.015**
Sex					
Male	ref				
Female	3.98	0.85	2.29	5.66	**<0.01**
Marital status					
Currently married	ref				
Currently not married	1.64	0.78	0.11	3.17	**0.036**
Level of education					
No formal	ref				
Primary	−5.26	5.79	−16.63	6.10	0.364
Secondary	−2.51	5.29	−12.90	7.86	0.634
Tertiary	−0.46	5.26	−10.79	9.85	0.929
Cadre					
Nurse	ref				
Community health officer	−1.57	2.18	−5.86	2.71	0.472
CHEW	0.31	2.12	−3.85	2.71	0.472
JCHEW	1.02	2.39	−3.66	5.71	0.668
*Others	−2.03	2.07	−6.08	2.02	0.327
Mode of payment for work done on vaccination					
Both cash and digital	ref				
Digital only	−2.26	0.83	−3.86,	−0.67	**0.005**
Years of work experience					
<2	ref				
2–5	−1.32	1.71	−4.68	2.04	0.442
6–10	−1.25	1.70	−4.60	2.08	0.461
More than 10	−1.75	1.72	−5.12	1.62	0.309
Region of residence					
North Central	ref				
North West	10.67	0.95	8.80	12.54	**<0.001**
South East	19.81	2.18	15.53	24.09	**<0.001**
South-South	14.26	1.01	12.26	16.25	**<0.001**
South West	11.76	1.07	9.65	13.86	**<0.001**
Able to use the internet					
No	ref				
Yes	4.10	1.04	2.06	6.15	**<0.01**

Bold values indicate statistically significant results (p < 0.05).

* Others were social workers, health assistant/technician, community mobilisers, public health personnel/health educator, volunteer workers, environmental health officer/attendant.

CHEW, community health extension workers; JCHEW, junior community health extension workers.

### Predictors of overall health workers’ perceived motivation, satisfaction and performance during digital payment

The adjusted LMM of the overall health workers’ perceived motivation, satisfaction and performance is shown in table 5. The model selection criteria included Akaike Information Criterion (AIC)=5294.61; Bayesian Information Criterion (BIC)=5349.69; −2LogL=−2635.30. Being female (β=1.83; 95% CI=0.28 to 3.38), not being married (β=1.43; 95% CI=0.01 to 2.86), able to use the internet (β=1.87; 95% CI=0.02 to 3.72) and residing in North West (β=9.64; 95% CI=7.66 to 11.63), South East (β=20.01; 95% CI=15.63 to 24.40), South-South (β=13.58; 95% CI=11.47 to 15.68) and South West zones (β=11.12; 95% CI=8.95 to 13.29), were significant predictors of health workers’ overall motivation, satisfaction and performance (table 5).

**Table 5:** Adjusted linear mixed model of overall health workers’ perceived motivation, satisfaction and performance during digital payment

		95% CI		
	**Adjusted coefficient**	**Lower**	**Upper**	**P value**
Location				
Rural	ref			
Urban	0.09	−1.32	1.51	0.896
Sex				
Male	ref			
Female	1.83	0.28	3.38	**0.02**
Marital status				
Currently married	ref			
Currently not married	1.43	0.01	2.86	**0.049**
Mode of payment				
Both cash and digital	ref			
Digital only	−0.40	−1.86	1.05	0.586
Region				
North Central	ref			
North West	9.64	7.66	11.63	**<0.01**
South East	20.01	15.63	24.40	**<0.01**
South-South	13.58	11.47	15.68	**<0.01**
South West	11.12	8.95	13.29	**<0.01**
Able to use the internet				
No	ref			
Yes	1.87	0.02	3.72	**0.047**

Bold values indicate statistically significant results (p < 0.05).

## Discussion

This study examined the influence of DFS on perceived motivation, satisfaction and performance of health campaign workers in Nigeria. The study showed that using digital payment systems during vaccination campaigns was associated with health workers’ self-reported motivation, job satisfaction and performance. Prior research supported this finding, indicating that the presence of digital financial options can address some of the financial obstacles experienced by health workers when receiving payments for their services.^30 31^ Also, previous research revealed that digital payment systems provided a convenient and secure method that can be used to disburse salaries, incentives and other monetary benefits.^21 30 31^ The more secure payment provided by electronic payments can lead to improved financial well-being of health workers, which can result in increased job satisfaction and motivation, thereby improving their overall job performance.^21^

Traditional methods of payments, such as physical paychecks or cash payments, are fraught with delays, inconsistencies and opportunities for corruption.^30 32^ A recent study investigated the effectiveness of implementing a mobile money payment strategy to ensure prompt and comprehensive compensation for frontline health campaign workers engaged in the polio vaccination efforts in Africa. The study highlighted the importance of the use of electronic payment systems to remunerate these important workers.^7^ Also, the study found that DFS provided the opportunity for committed health systems to improve efficiency and efficacy of health campaigns in resource-constrained countries.^7^ Health workers who had reliable and secure channels for receipt of payments were less likely to be distracted about concerns on payment modalities and therefore more likely to focus on their work, consequently leading to increased satisfaction, better performance and improved programme outcomes.^33 34^ However, it is important to note that improved work outcomes may also be influenced by other factors including training, work conditions, other job benefits, career fulfilment and management practices.^22 23 35 36^ Also, the provision of adequate tools and equipment, transportation, communication, appreciation of work done, good performance evaluation and work schedule influence health workers’ performance and motivation.^36^ Hence, it is essential for administrators of health campaigns to use a comprehensive approach that combines DFS with other interventions, in order to optimise healthcare worker motivation, satisfaction and performance.^37 38^ This conclusion further emphasises how full adoption of the digital payment for health campaigns may lead to improved transparency and financial accountability, which can result in better motivation, satisfaction and performance among campaign health workers.

Our findings showed that health campaign workers reported experiencing improvement across the different constructs of motivation, satisfaction and performance including general motivation, job satisfaction, intrinsic job contentment, organisational dedication, conscientiousness, punctuality and overall work performance. Comparison of perceived motivation, satisfaction and performance scores for digital payments and cash disbursements showed that the health workers had greater satisfaction and were more effective with the use of electronic payments. Despite these findings, some challenges with the implementation of DFS in Nigeria have been reported. These challenges include: inadequate internet network infrastructure and coverage, high costs of DFS operations, internet/digital registration challenges and low literacy levels among certain cadres of campaign workers.^38^ Limited skills of the campaign workers and programme managers; cumbersome verification process, as well as use of paper-based documentation, have also been reported to hamper the use of DFS.^29^ Therefore, it is crucial to address both the administrative and technological challenges in order to overcome the problem of poor connectivity, improve the performance of DFS and maximise benefits of health campaigns. DFS managers need to seek support from policymakers in order to create a more enabling environment for DFS implementation in the healthcare sector.

Sociodemographic factors were associated with health workers’ perceived motivation, satisfaction and performance in the context of payment methods. Marital status and sex (female) were associated with higher perceived motivation, satisfaction and performance among health workers. Similarly, geographical zone was associated with health workers’ perceived motivation, satisfaction and performance. This may be due to the regional differences in the availability of the critical infrastructure that supports the use of DFS in Nigeria. There are disparities in literacy levels, mobile phone ownership and internet access between the southern and northern regions of the country.^39^ Hence, interventions need to take these regional differences into consideration, as they strive to improve the experiences of health campaign workers in Nigeria.

Additionally, our findings indicate that among health workers who were paid through DFS, those who had experienced both cash and digital payments reported significantly higher levels of perceived motivation, satisfaction and performance compared with those who had only received payments through digital means. This finding could be explained by the aforementioned challenges commonly associated with digital payment systems in low-resource settings. A previous research^39^ documented some other barriers to effective digital payments, including failure to process payment due to incorrect beneficiary account details and the service fees or transaction charges associated with digital transfers. Such challenges can undermine workers’ confidence in the digital system, making some prefer the immediacy and perceived reliability of cash payments. Despite these obstacles, digital payment systems appear to be more advantageous than cash, offering improved transparency, security and accountability in financial transactions. To optimise the benefits of DFS for health workers’ compensation, it is imperative to address the systemic and infrastructural challenges that continue to limit its effectiveness. Strengthening digital payment infrastructure, ensuring accurate documentation of beneficiary details, reducing transaction costs and expanding banking access to rural populace are critical steps towards maximising the potential of DFS to strengthen the health workforce performance and motivation.

Furthermore, it is also important to implement interventions aimed at improving digital literacy among healthcare workers. Health workers who were proficient in the use of the internet experienced better perceived motivation, satisfaction and performance. This highlights the importance of digital training initiatives to ensure that health workers have the skills to navigate electronic payments systems including the use of e-wallets. While cash payments were associated with higher perceived motivation, satisfaction and performance in urban settings, the same was not observed with digital payment methods. This calls for further research on the contextual factors that may shape health workers’ experiences in these settings, as well as the potential role of payment modalities in influencing their choices.

### Study strengths and limitations

The strength of this study is that it is one of the first studies that explored and compared digital and cash payments of healthcare workers using a national/population based approach. Our limitation lies in the cross-sectional nature of the study design. Hence, we could only test association and not causation. Despite this, our results would guide policy makers on how to promote healthcare workers’ motivation and performance using DFS. Additionally, an important limitation relates to the validity and precision of our estimates. While health campaigns are implemented across all Nigerian states with similar operational structures including modes of payment, we did not apply design-adjustment variables such as sampling weights or account for clustering by location (eg, state or LGA). Although this is unlikely to have introduced substantial systematic bias, the absence of such adjustments may affect the representativeness and generalisability of our findings. Readers and policymakers should interpret the results with this limitation in mind. Future studies employing probability-based sampling and design-adjusted analyses would be useful to validate and extend these findings.

## Conclusion

The adoption of digitised payment methods was associated with higher perceived motivation, satisfaction and performance among health campaign workers. Workers reported better outcomes when digital payment methods were used compared with traditional cash payments. However, to fully realise the potential of DFS, challenges such as inadequate network infrastructure, high operational costs, high service charges and limited digital literacy among workers need to be addressed. These barriers hinder the efficiency and effectiveness of the payment system.

## Supplementary material

10.1136/bmjgh-2024-018384 online supplemental file 1

## Data Availability

All data relevant to the study are included in the article or uploaded as supplementary information.
